# Identifying the Effects of Reactive Oxygen Species on Mitochondrial Dynamics and Cytoskeleton Stability in *Dictyostelium discoideum*

**DOI:** 10.3390/cells10082147

**Published:** 2021-08-20

**Authors:** Evan Downs, Amber D. Bottrell, Kari Naylor

**Affiliations:** Department of Biology, University of Central Arkansas, Conway, AR 72035, USA; edowns3@cub.uca.edu (E.D.); abottrell1@cub.uca.edu (A.D.B.)

**Keywords:** mitochondrial dynamics, fission, fusion, motility, ROS, microtubules

## Abstract

Defects in mitochondrial dynamics, fission, fusion, and motility have been implicated in the pathogenesis of multiple neurodegenerative diseases, including Parkinson’s disease, Alzheimer’s disease, Huntington’s disease, and Charcot–Marie–Tooth disease. Another key feature of neurodegeneration is the increase in reactive oxygen species (ROS). Previous work has shown that the cytoskeleton, in particular the microtubules, and ROS generated by rotenone significantly regulate mitochondrial dynamics in *Dictyostelium discoideum.* The goal of this project is to study the effects of ROS on mitochondrial dynamics within our model organism *D. discoideum* to further understand the underlying issues that are the root of neurodegenerative diseases such as Alzheimer’s disease and Parkinson’s disease. We chose three likely ROS inducers, cumene hydroperoxide, hydroxylamine hydrochloride, and Antimycin A. Our work demonstrates that alteration of the microtubule cytoskeleton is not required to alter dynamics in response to ROS and there is no easy way to predict how mitochondrial dynamics will be altered based on which ROS generator is used. This research contributes to the better understanding of the cellular mechanisms that induce the pathogenesis of incurable neurodegenerative diseases with the hope that it will translate into developing new and more effective treatments for patients afflicted by them.

## 1. Introduction

Parkinson’s disease affects over four million people worldwide and in the United States approximately 60,000 new cases are diagnosed every year, making it one of the most common neurodegenerative diseases worldwide [1]. Neurons are reliant upon mitochondrial dynamics, fission, fusion, and motility, as well as mitochondrial localization within the cell to properly function [2,3]. Defects in these equilibriums are associated with several neurodegenerative diseases [4,5,6,7]. Another key feature of neurodegeneration is the increase in reactive oxygen species (ROS) [8]. The goal of this project was to study the effects of ROS on mitochondrial dynamics within our model organism *Dictyostelium discoideum* to further understand these underlying issues that are associated with neurodegenerative diseases. *D. discoideum* is a well-established model system for studying mitochondrial dynamics [9,10] and mitochondrial disease including the cellular aspects of Parkinson’s disease in the presence of rotenone [11,12,13,14].

Mitochondria are mobile organelles that move throughout the cell, continuously dividing and joining through processes known as fission and fusion [5,15]. Fusion is the process by which stressed or damaged mitochondria join together as a form of stress mitigation. Mitochondria that have been damaged by environmental factors, developed mutations within their genome, or that are experiencing oxidative stress can merge with healthy mitochondria within a cell as a form of compensation in which the newly formed mitochondria shares components of the two previously independent organelles [16]. This process serves to maximize the oxidative capacity and to regulate the performance of individual mitochondria. Fission is another quality control process that occurs in equilibrium with fusion in which mitochondria split in order to create new mitochondria as well as to remove damaged fragments that are inhibiting the function of the organelle [16]. Microtubules are critical structures for these processes as well as being important in mitochondrial motility [10].

Mitochondrial motility is the ability of mitochondria to move within a cell and is often facilitated by cytoskeleton components including intermediate filaments, actin filaments, motor proteins, and in particular microtubules [6]. This movement is crucial to cellular processes, such as cell cycle progression and apoptosis that require the proper localization of mitochondria. Motility demands are especially high in elongated and polarized cells, including animal neurons, due to the length of their structure and specialized function [6].

Disruptions in mitochondrial fission, fusion, and motility have been implicated in the pathogenesis of multiple neurodegenerative diseases, including Parkinson’s disease, Alzheimer’s disease, Huntington’s disease, and Charcot–Marie–Tooth disease [5,6,7]. Although the production of ATP is the primary function of mitochondria, they are also involved extensively in a myriad of other cellular functions. Mitochondria associated with the endoplasmic reticulum are involved in calcium homeostasis which in turn can initiate apoptosis or improve oxidative metabolism [5]. Other mitochondria located within the pulmonary arterial system function as oxygen sensors by altering the production of ROS in response to fluctuating cellular oxygen levels which facilitates the redox regulation of ion channels, enzymes, and transcription factors. Defects in these complex pathways result in decreased metabolic efficiency and increased oxidative stress which are the hallmarks of diseased neurological cells [5].

The cytoskeleton, in particular the microtubules, play a key role in mitochondrial dynamics in *D. discoideum.* The disruption of microtubules has been shown to result in a drastic decrease in mitochondrial motility as well as fission and fusion events [10]. Recent work by our lab has also shown that rotenone (an inducer of Parkinson’s disease) increases mitochondrial velocity, inhibits fusion, increases ROS, and disrupts microtubule structures [11]. ROS are produced as byproducts of cellular metabolism and include molecules derived from molecular oxygen that include hydrogen peroxide (H_2_O_2_), superoxide anions (O_2_^−^), and hydroxyl radicals (^−^OH). These molecules play a role in apoptosis, immunological functions, and autophagy. If the production and elimination of ROS falls out of equilibrium, ROS accumulation and neurodegeneration can occur [7,17]. ROS are typically produced via complex I and complex III of the electron transport chain and if not dealt with, can lead to mitochondrial dysfunction, resulting in the neurological symptoms common within neurodegenerative diseases [18,19].

For this project, three likely ROS inducers were chosen and their effects on ROS production, mitochondrial dynamics and cytoskeleton stability in *D. discoideum* analyzed. Here, we chose to look specifically at the relationship between ROS and mitochondrial dynamics to determine whether mitochondrial dynamics are identically altered across different types of ROS inducers and whether the cytoskeleton is always involved in these changes. By using a simple model to study mitochondrial dynamics that clearly are associated with neurodegeneration and ROS imbalance [5,20,21], we can contribute to a greater understanding of the mechanisms behind the degradation of neurons. Finally, little work has been performed in *D. discoideum* studying the effects of ROS, and the ROS inducers presented here are ones that induce detectable ROS by dihydroethidium (DHE). Cumene hydroperoxide is an organic molecule and known oxidizing agent that affects complex III of the electron transport chain, thus decreasing ATP concentrations and mitochondrial membrane potential [22,23]. Hydroxylamine hydrochloride (HA) is an organic crystalline solid used in organic syntheses and photographic development. It was selected for this study due to its known ability to inhibit catalase thereby impeding hydrogen peroxide decomposition within the cell, which in turn increases the likelihood of oxidative damage to cellular components [24,25]. Antimycin A (AMA) is a known inducer of ROS in human pulmonary fibroblasts and functions by inhibiting succinate and NADH oxidases as well as by binding to complex III of the electron transport chain [26,27]. The inhibition of complex III results in disruption of the proton gradient across the inner mitochondrial membrane, leading to an overproduction of ROS within the cell [28,29].

Mitochondria possess highly efficient antioxidant systems which work to neutralize excess ROS production and prevent accumulation that can result in increased oxidative stress. When these systems are disrupted by environmental toxins, mutations, or cellular aging, the resulting oxidative stress leads to an alteration in mitochondrial redox homeostasis [30]. Long-lived non-mitotic cells such as neurons are particularly susceptible to irreparable damage as a result of ROS accumulation. This accumulation has been shown to disrupt redox homeostasis within these cells, which has been linked to the pathogenesis of neurodegenerative diseases such as Alzheimer’s disease and Parkinson’s disease [30,31]. In this work, we build on the results of Chernivec et al., 2018 and analyze the relationship between ROS, mitochondrial dynamics and microtubule structure [11].

## 2. Materials and Methods

### 2.1. Strain Growth

The AX4 (wild-type) strain of *Dictyostelium discoideum* was obtained from Bill Loomis via Dicty Stock Center (www.dictybase.com (accessed on 12 August 2021)). AX4 cells were cultured in liquid HL5 media [32,33] containing streptomycin (300 μg/mL) and ampicillin (150 μg/mL) at 22 °C with shaking at 125 rpm.

### 2.2. ROS Induction

Log-phase cells in HL5 were exposed to varying concentrations of cumene hydroperoxide (0–1.5 mM) for 30 min, hydroxylamine hydrochloride (HA) (0–5 mM for) for 1 h, or Antimycin A (AMA) (0–50 μM) for 1 h with shaking at 22 °C, then cells underwent ROS measurement, fixation for immunofluorescence, or confocal imaging. The vehicle control for cumene and AMA was ethanol, and the vehicle for HA was water.

### 2.3. ROS Assay

Log-phase cells were pelleted and resuspended in SS6.4 Buffer Solution to 6 × 10^6^ cells/mL for cumene assays or 2.68 × 10^6^ to 2.75 × 10^6^ cells/mL for HA and AMA assays. Fluorescence values were normalized to cell number for each assay. A volume of 50 μL of treated cells was added to appropriate wells of a black 96-well plate in triplicate. A volume of 50 μL of 60 μM dihydroethidium (DHE) (Invitrogen, Thermo Fisher Scientific, Grand Island, NY, USA) was added to each well and fluorescence was measured using BioTek’s Synergy H1. Cells were excited at 522 nm and emission was measured at 605 nm for 10 min, with a reading taking place every 30 s. Cumene readings over the 10 min were averaged, then a mean for the triplicate readings was calculated. For HA and AMA, only the first triplicate readings were averaged. Assays were carried out a minimum of 5 times. Statistical analysis was performed using GraphPad Prism version 6.07 for Windows (GraphPad Software, La Jolla, CA, USA, www.graphpad.com (accessed on 12 August 2021)). One-way ANOVA with Tukey’s post hoc was used.

### 2.4. Live-Cell Imaging

Log-phase cells were washed one time with Lo-Flo media (Formedium, Hunstanton, Norfolk, UK), resuspending to original volume in Lo-Flo. Mitotracker CMXRos (Invitrogen, Thermo Fisher Scientific, Grand Island, NY, USA) at a final concentration of 0.1 μM was added and cells were incubated for 4 h at 22 °C with shaking at 125 rpm. Cells were washed 3 times with Lo-Flo and placed in Nunc Lab-TekII 4-well chambered cover glass for imaging. Cells were treated with 0, 0.3 or 0.6 mM cumene for 30 min prior to imaging, 0, 3, or 5 mM HA for 60 min prior to imaging, or 0, 30, or 50 μM AMA 60 min prior to imaging. Cells were imaged with Nikon A1R confocal on a Ti2-E inverted microscope (Nikon, Melville, NY, USA). Live-cell imaging settings included resonance scanning of a single section with a pinhole of 45.98, and movies were obtained for 2 min with 0.065 s timepoints. Cells were imaged on a minimum of 3–4 separate occasions with approximately 5 time-lapse images obtained each time.

### 2.5. Quantification of Fission and Fusion

Image analysis of fission and fusion was performed using NIS Elements AR 5.20.02 (Nikon, Melville, NY USA) similarly to past work [9,10,11,34]. Fission was determined by a single organelle splitting into two organelles. Fusion was quantified by two mitochondria nearing each other for multiple frames and then fusing into one organelle. The rates of fission and fusion were determined by calculating the average number of events/min./cell. Images were denoised and ≥30 cells were analyzed. The Kruskal–Wallis test with Dunn’s post hoc, at an alpha level of 0.05 statistical analysis, was performed using GraphPad Prism version 6.07 for Windows (GraphPad Software, La Jolla, CA, USA, www.graphpad.com (accessed on 12 August 2021)).

### 2.6. Quantification of Motility

Using NIS Elements AR 5.20.02 (Nikon, Melville, NY USA) live-cell images were denoised and then kymographs by line were created, similarly to past work [10,11,34]. A curvilinear line was drawn across three vertical areas of a cell, ensuring a minimum of 3 mitochondria were captured. On the kymograph, a simple line was drawn across every visible mitochondria until direction changed and then a new line was drawn (50–460 lines/measurements per kymograph). The slope of each line is the velocity of movement. A total of 30 cells across 3–4 imaging experiments were analyzed, and slopes were averaged for each cell, ignoring zero slopes. The velocity in μm/s for each cell was then averaged across the 30 cells. To calculate % motility, the number of velocities above 0 μm/s was divided by the total number of velocities measured. Thus, % motility was not calculated per mitochondria but essentially the number of times organelles were not moving. To determine differences in velocity, GraphPad Prism version 6.07 for Windows (GraphPad Software, La Jolla, CA, USA, www.graphpad.com (accessed on 12 August 2021)), the Kruskal–Wallis test with Dunn’s post hoc test or one-way ANOVA with Tukey’s post hoc test was performed depending on whether the data were parametric. An alpha level of 0.05 was used.

### 2.7. Quantification of Microtubule Stability

**Figure 1:**
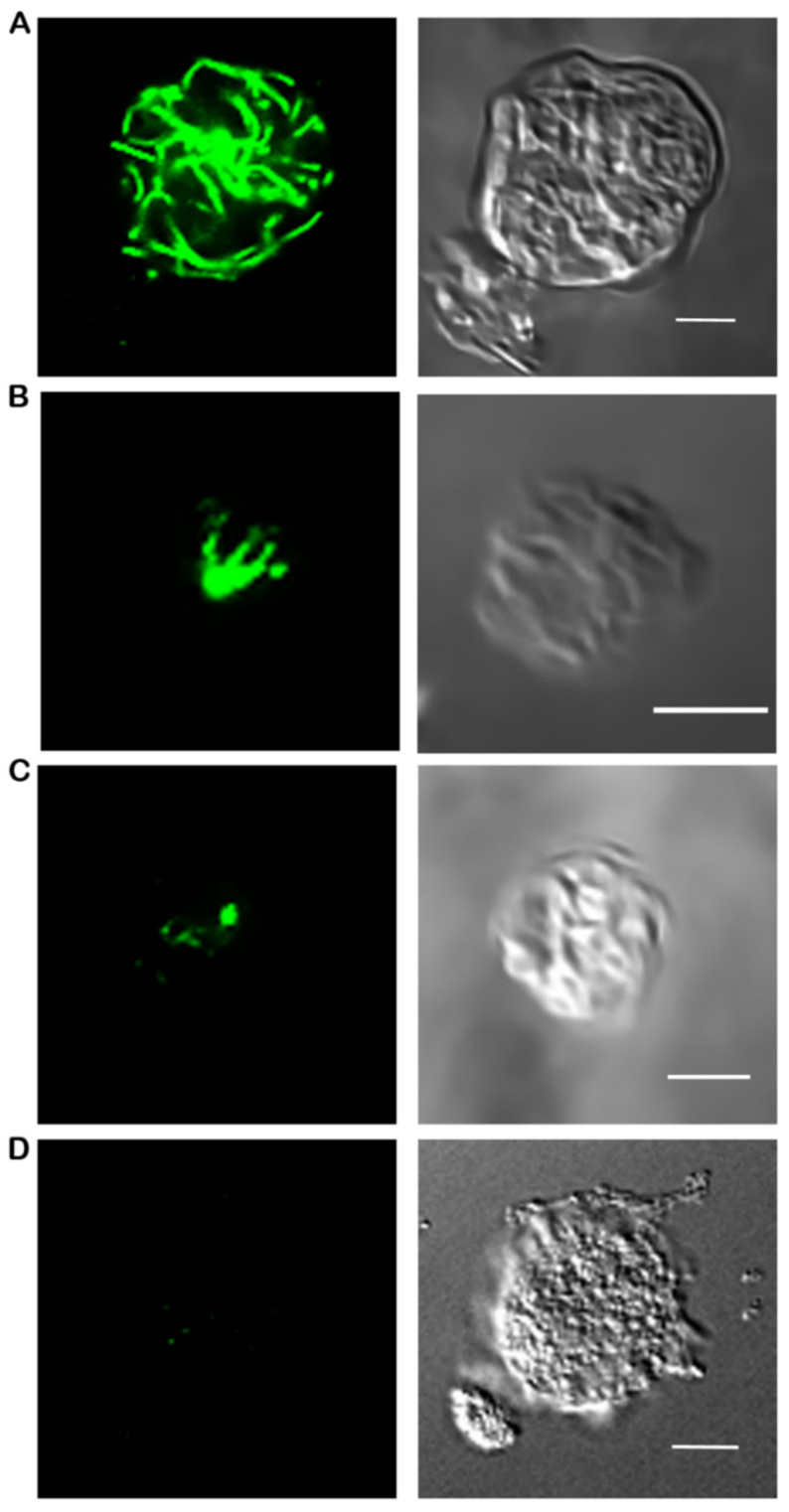
Microtubule Morphologies. (**A**) Fully intact microtubule structures with numerous tubules reaching toward the plasma membrane are characterized as intact. (**B**) A cytoskeleton that demonstrates an astral shape but does not extend to the plasma membrane is designated as partial. (**C**) Spot morphologies are where only the microtubule organizing center is present and (**D**) dead cells are when there is no detectable fluorescence (as shown here) or the plasma membrane is obviously no longer intact (data not shown). Scale bar is 5 μM.

Immunofluorescence imaging was utilized to determine the morphology of microtubules. Log-phase cells were treated with 0, 3, or 5 mM of HA, or 0, 30, or 50 μM for AMA for 30 min shaking at 22 °C (log-phase cells were treated with cumene only on coverslips, see below). Then, 15 mL of cells were washed twice with Lo-Flo and resuspended to 4–8 × 10^6^ cells/mL. Further, 0.5 mL of washed cells were added directly to a coverslip in a 6-well plate and incubated for 30 min at room temperature with appropriate treatment (0, 0.3, or 0.6 mM for cumene; 0, 3, or 5 mM for HA, or 0, 30, or 50 μM for AMA). Cells were washed two times with 10 mM MES-NaOH, then fixed with 3% paraformaldehyde pH 6.0 for 30 min. The cells were quenched with 100 mM glycine in 1 × PBS for 5 min then permeabilized with 0.02% Triton X-100 for 5 min. After three quick 1 × PBS washes, cells were incubated in 0.045% fish gelatin and 0.5% BSA in 1 × PBS (PBG) for 1 h at room temperature. Mouse anti-tubulin (12G10, Developmental Studies Hybridoma Bank, Iowa City, IA, USA) diluted 1:150 in PBG solution was added to the coverslips and incubated at 4 °C 12–18 h. Three 5 min washes with 1 × PBS were performed and the secondary antibody was added, AlexaFluor 488 goat alpha mouse IgG, (Life Tech, A11001, Thermo Fisher Scientific Grand Island, NY, USA) 1:250 in PBG solution for 1 h at 4 °C. The coverslips were kept in the dark from this point forward. Three final 5 min washes in 1 × PBS were performed then coverslips were mounted to glass slides with SlowFade Diamond (Invitrogen, Thermo Fisher Scientific Grand Island, NY, USA) and stored in the dark at 4 °C. Immunofluorescence imaging was performed on a Nikon A1R confocal on a Ti2-E inverted microscope (Nikon, Melville, NY, USA), settings included Galvano scanning with a pinhole of 26.82. A total of 61 z-sections, 0.1 um thick, were obtained. Image analysis was performed using NIS Elements AR 5.20.02 (Nikon, Melville, NY USA). Microtubules were categorized into intact, partial, spot, or dead based on microtubule morphology for at least 50 cells per treatment by two independent researchers (Figure 1). Intact refers to a complete microtubule complex extending throughout the cell, partial means less a complete complex and shorter microtubule filaments that do not reach periphery of the cell, spot indicates only the center of the microtubule organizing center is present with no radiating tubules, and dead represents cells where microtubules were depolymerized due to cellular death prior to fixation. Statistical analysis was performed using Chi Square analysis in GraphPad Prism version 6.07 for Windows (GraphPad Software, La Jolla, CA, USA, www.graphpad.com (accessed on 12 August 2021)).

## 3. Results

### 3.1. Cumene Hydroperoxide Decreases Mitochondrial Velocity and Disrupts the Cytoskeleton

**Figure 2:**
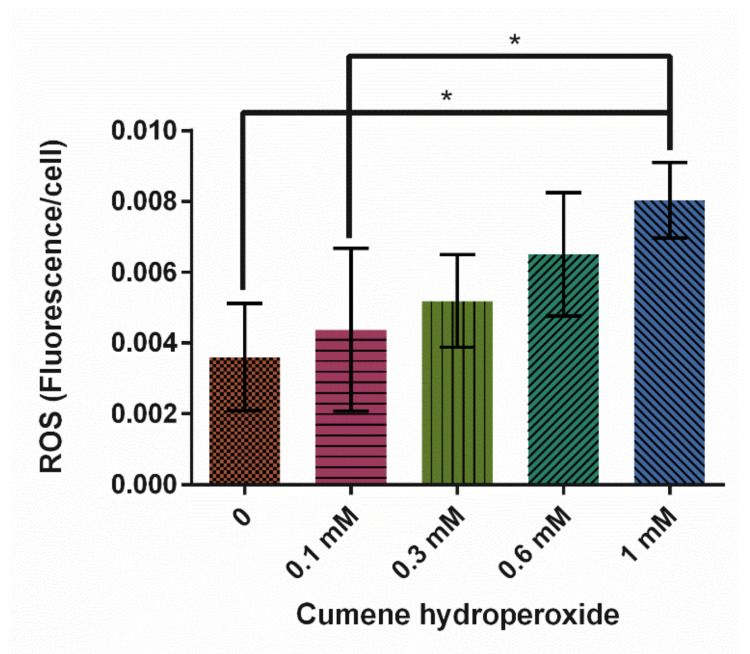
Effect of cumene hydroperoxide on average reactive oxygen species (ROS) levels in *D. discoideum* ± SE (*N* = 5). Cumene significantly increased ROS levels as indicated by * (*p* = 0.0116).

Cumene hydroperoxide has been used in previous studies as a positive ROS control and to induce oxidative stress in *Dictyostelium discoideum* [11,35,36]. In this study, cumene was shown to significantly increase ROS production (Figure 2, *p* = 0.0116). A total of 1 mM cumene induced the highest levels of ROS and was on average 123% higher than the 0 mM control.

**Figure 3:**
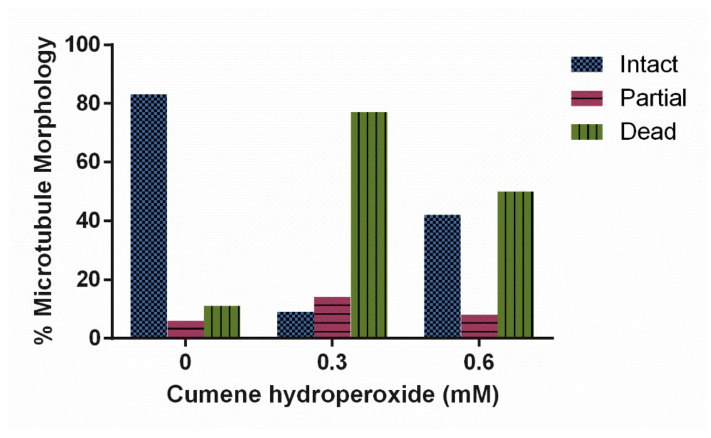
Effect of cumene hydroperoxide on microtubule morphology in *D. discoideum.* Cumene significantly decreased microtubule stability (n > 50 cells, *p* < 0.0001).

Immunofluorescence analysis was used to determine microtubule morphology within cells treated with 0, 0.3 and 0.6 mM concentrations of cumene hydroperoxide. Increasing cumene concentration significantly decreased the stability of microtubules in *D. discoideum* (Figure 3, *p* < 0.0001). A total of 88% of the untreated cell’s microtubule structures were intact in contrast to only 21% of 0.3 mM and 53% of 0.6 mM cumene-treated cells (Figure 3). Cumene increased AX4 mortality at all concentrations tested—0 mM yielded 10% dead cells, 0.3 mM 28%, and 0.6 mM 39%.

**Figure 4:**
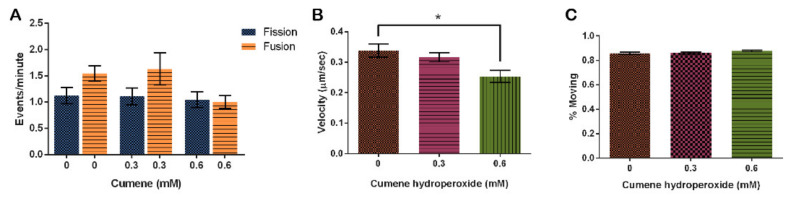
Effect of cumene hydroperoxide on mitochondrial dynamics. (**A**) Fusion trends faster in vehicle control and 0.3 mM cumene, but cumene does not significantly alter fission and fusion (n = 30 cells, *p* = *0*.0896). (**B**) Cumene significantly decreases mitochondrial velocity but (**C**) has no effect on the number of mitochondria moving (n = 30 cells, *p*= 0.0062 and 0.1669, respectively). Columns are averages, error bars represent the standard error and * indicates significance.

Cumene hydroperoxide did not have a significant effect on the rate of mitochondrial fission and fusion in this study. However, fusion rates did trend higher at the control and 0.3 mM concentrations (Figure 4A, *p* = 0.0896). Fission rates remained relatively stable throughout all concentrations tested, while fusion at the 0.6 mM concentration decreased to match fission (Figure 4A).

Finally, we analyzed the effect of cumene on mitochondrial motility. Mitochondria on average move 0.34 ± 0.02 μm/s when treated with the vehicle control, EtOH. This speed decreased with the addition of cumene (Figure 4B, *p* = 0.0062), decreasing by 6.3% with 0.3 mM cumene and 36% with 0.6 mM cumene from the control (Figure 4B). Though the rate significantly slows down, the percentage of mitochondria actually moving remained the same (Figure 4C, *p* = 0.1669).

In summary, cumene increased ROS in *D. dictyostelium*, destabilized the microtubules, has no significant effect on mitochondrial fission, fusion, and percent motility, but did significantly decrease the velocity at which the mitochondria move.

### 3.2. Hydroxylamine Hydrochloride Does Not Significantly Alter Dynamics

**Figure 5:**
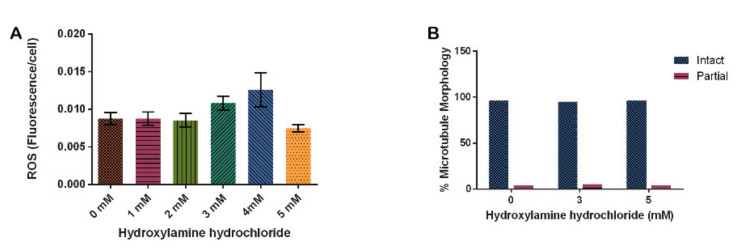
Effect of hydroxylamine hydrochloride on in vivo reactive oxygen species production and microtubule stability in *D. discoideum.* (**A**) HA increases ROS at 3 and 4 mM but this is not significant (*N* = 5, *p* = 0.3120). (**B**) HA has no effect on microtubule morphology in *D. discoideum* (n > 50, *p* = 0.9495). Columns are averages and error bars represent the standard error.

To determine the effect of hydroxylamine hydrochloride in our model system on ROS levels, we again used DHE to detect ROS. Our results indicate that ROS increased at 3 mM HA by 23% and 4 mM HA by 44% though these increases were not significant (*p* = 0.1320). At 5 mM, ROS decreased similarly to the untreated control (Figure 5A). Analysis of microtubule structure in cells treated with HA indicated that HA had no effect on microtubules (Figure 5B, *p* = 0.9495).

**Figure 6:**
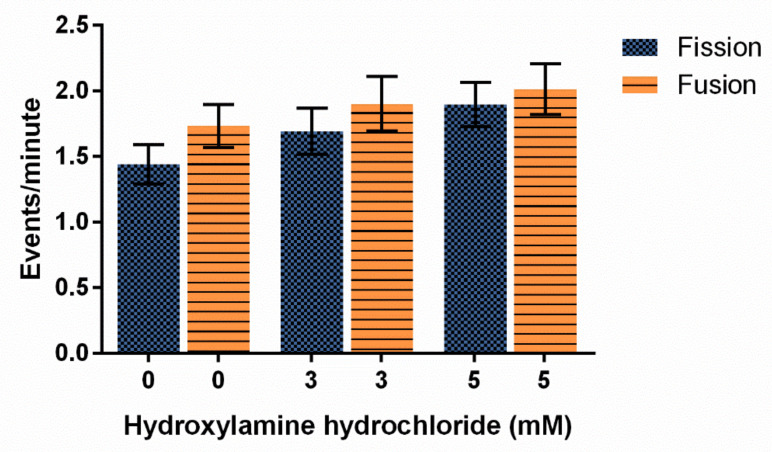
Effect of hydroxylamine hydrochloride on mitochondrial fission and fusion frequency in *D. discoideum.* Increasing HA does not significantly alter average ± SE fission and fusion events, though these rates trend faster with more HA (n = 34 cells for each treatment, *p* = 0.2450).

Hydroxylamine hydrochloride also had no significant effect on fission and fusion frequencies at the concentrations tested, though fission and fusion events did trend upwards as HA concentrations increased with the highest rates of both fission and fusion occurring at the 5 mM concentration (Figure 6, *p* = 0.2450).

**Figure 7:**
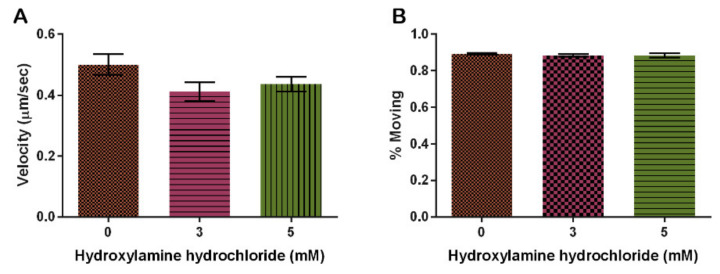
Effect of hydroxylamine hydrochloride on mitochondrial motility. (**A**) Increasing HA does not significantly alter velocity though it decreases slightly with increasing concentrations of HA. (**B**) Increasing HA does not alter the percentage of mitochondria moving (n = 30, *p* = 0.2670 and 0.1519, respectively). Columns are averages and error bars represent the standard error.

Finally, we show that HA had no effect on the amount of mitochondria moving at any one time (Figure 7B, *p* = 0.1519). The 3 mM HA concentration decreased the rate of mitochondria movement by 22% from the vehicle control and 5 mM HA concentration decreased by 30% from the control, but it is not significant (Figure 7A, *p* = 0.2670).

The conclusion of HA treatments on *D. discoideum* cells is that HA did not significantly alter any measured effects in this study. Trends indicate that HA increased ROS, increased fission and fusion, decreased mitochondrial velocity, and showed no changes to microtubule structure or percentage of mitochondria moving.

### 3.3. Antimycin a Decreases Fission and Fusion but Increases Mitochondrial Velocity

**Figure 8:**
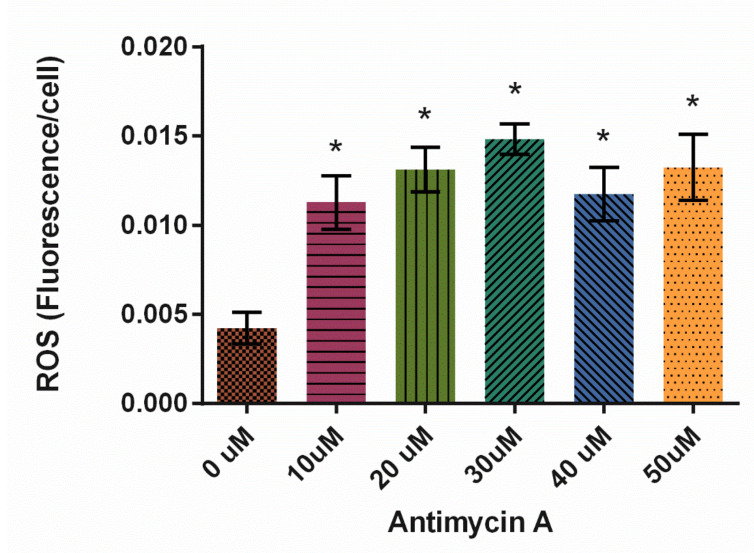
Effect of Antimycin A on average reactive oxygen species (ROS) levels in *D. discoideum* ± SE (*N* = 6). AMA significantly increased ROS levels compared to control, as indicated by * (*p* < 0.0001).

In this study, Antimycin A significantly increased ROS production at all concentrations tested, with 30 μM resulting in the highest levels of ROS (Figure 8, *p* < 0.0001).

**Figure 9:**
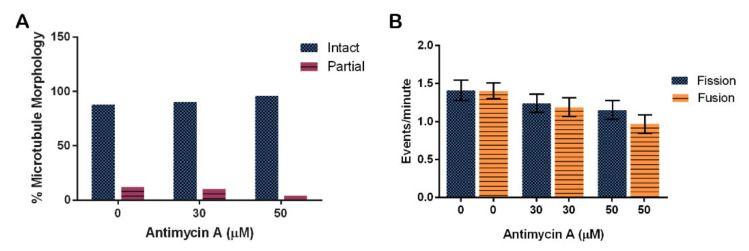
Effect of Antimycin A on microtubule morphology and mitochondrial fission and fusion in *D. discoideum.* (**A**) AMA did not significantly affect microtubule stability (n > 50 cells, *p* = 0.2846), nor (**B**) fission and fusion rates (n = 46 cells, *p* = 0.0516), though these rates trend faster with less AMA. Columns are averages and error bars represent the standard error.

Analysis of microtubule structure in the presence of AMA using immunofluorescence showed that AMA had no effect on tubulin structures (Figure 9A, *p* = 0.2846). The trend suggests that perhaps AMA stabilizes microtubule structures. Quantification determined that 88% of control cells, 90% of 30 μM AMA-treated cells, and 96% of 50 μM AMA-treated cells have intact microtubule structure.

Fission and fusion events per minute were quantified in cells at different concentrations of AMA. There were on average 1.4 ± 0.2 fission and 1.4 ± 0.1 fusion events in cells treated with the vehicle control EtOH. Fission rates decreased by 17% for the 30 μM treatment and by 26% for the 50 μM treatment when compared to control rates. Fusion rates also decreased in the 30 and 50 μM treatments by 21% and 44%, respectively (Figure 9B, *p* = 0.0516).

**Figure 10:**
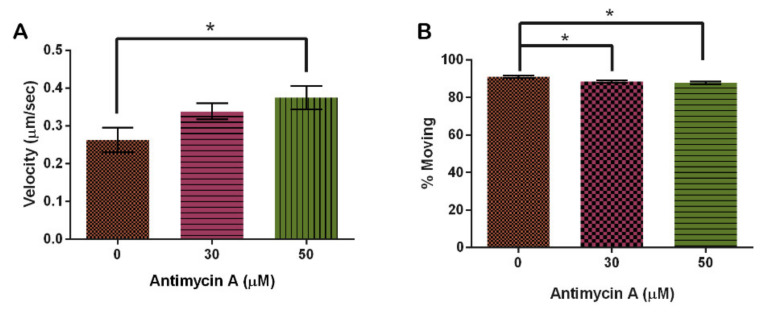
Effect of Antimycin A on mitochondrial motility. (**A**) AMA significantly increases mitochondrial velocity and (**B**) has a significant effect on the number of mitochondria moving (n = 30 cells, *p* = 0.0436 and 0.0041, respectively). Columns are averages, error bars represent the standard error and * indicates significance.

Analysis of motility in the presence of AMA indicated that velocity increased with rising AMA concentration. In the vehicle control, mitochondria moved 0.26 ± 0.03 μm/s, increasing by 24% with 30 μM and 29% with 60 μM AMA from the control group (*p* = 0.0436) (Figure 10A). Interestingly, increasing AMA decreased the amount of movement of mitochondria even though velocity increased (*p* = 0.0041) (Figure 10B).

Finally, AMA has no effect on microtubule organization similar to HA. However, it decreased fission and fusion rates while simultaneously increasing organelle velocity. AMA was significantly increased ROS levels at every concentration tested suggesting it’s potential to efficiently induce ROS production in future *D. discoideum* studies.

## 4. Discussion

In our study, all three treatments, cumene, HA, and AMA, induced ROS but only cumene and AMA did so significantly (Figure 2, Figure 5A and Figure 8). Cumene is the only chemical tested that significantly affected microtubule morphologies (Figure 3, Figure 5B and Figure 9A). No ROS inducer significantly altered mitochondrial fission and fusion but all three did alter these dynamics slightly. Cumene decreased fusion from the control but did not alter fission rates, HA increased both fission and fusion, while AMA decreased the number of fission and fusion events (Figure 4A, Figure 6 and Figure 9B). These chemicals alter mitochondrial motility in different manners as well. Cumene and HA decreased the speed at which the mitochondria move while AMA increased mitochondrial velocity and decreased the percent of organelles moving (Figure 4B,C, Figure 7 and Figure 10). This work indicates that the presence of ROS alone does not alter mitochondrial dynamics and therefore either the type of ROS, the additional effects caused by the ROS inducing chemicals, or the method of ROS creation appears to play a role in altering mitochondrial dynamics.

Cumene is known to inhibit mitochondrial respiration and alters the function of complex III of the electron transport chain, resulting in superoxide formation [22,23]. Cumene does not appear to alter complex I directly but in conjunction with inhibition of complex III, complex I activity is altered [22]. Cumene likely also increases ROS through mechanisms not requiring oxygen [22]. Hydroxylamine hydrochloride causes ROS generation by inhibiting catalase enzymes, resulting in an increase in H_2_O_2_ [25,37,38]. Antimycin A also inhibits complex III, specifically through the ubiquinol oxidation center as well as inhibiting the mitochondrial multiple conductance channel, resulting in superoxide formation [19,26,27,29].

Looking at the results from this study, we can see that inhibition of complex III by cumene or AMA does not result in a similar effect on mitochondrial dynamics. Cumene decreased fusion and decreased mitochondrial velocity while AMA slightly decreased both fission and fusion and significantly increased velocity. While HA increases ROS through the inhibition of catalase rather than the electron transport chain, it does increase mitochondrial fission and fusion, the opposite of cumene and AMA, but there is no obvious pattern in terms of mitochondrial velocity Thus, we suggest that ROS generation via inhibition of the electron transport chain decreases fission and fusion but does not predict the effect on mitochondrial velocity, supported by Chernivec et al., as described below [11].

What about the effect on the cytoskeletal structure? It is well known that rotenone induces ROS and alters microtubule organization [39,40]. Rotenone inhibits complex I activity of the electron transport chain creating superoxides and H_2_O_2_ [18,41]. Recently we published the results of rotenone on *D. discoideum* dynamics [11]. We showed that rotenone disrupts the *D. discoideum* tubulin cytoskeleton, which cannot be returned to normal by the addition of reducing agents such as vitamin C [11]. Rotenone decreases fusion, which can be reversed by the addition of vitamin C, and it increases mitochondrial velocity with no effect on the number of mitochondria moving [11]. Here, we compare these results with the current study to identify the relationship between mitochondrial dynamics, microtubules and ROS.

ROS inducers that disrupt the *D. discoideum* microtubule cytoskeleton are rotenone and cumene, while AMA slightly stabilizes. Cumene, AMA and rotenone alter fission and fusion similarly, while cumene decreases velocity when rotenone and AMA increase it. This suggests that cytoskeletal disruption, which is known to alter mitochondrial dynamics [10] is not the only factor at play leading to these effects. We conclude that ROS alters mitochondrial dynamics, and these alterations do not require a change in microtubule organization.

So why the various alterations to mitochondrial dynamics? Why do they not cause the exact same effect across all of the inducers? ROS inducers do not just generate ROS. For example, rotenone has clearly been shown to bind to tubulin [40,42]. Cumene decreases cytosolic calcium levels and disrupts membranes [23]. In general, ROS function as signaling molecules sometimes directly inhibiting complex I of the electron transport chain [43,44,45], inducing transcription factors such as Nf-κB and HIF-1, as well as increasing antioxidant activity and regulating many other proteins. In terms of mitochondria, ROS have been shown to induce additional fragmentation of mammalian cells with Mfn2 mutations significantly altering mitochondrial quality control [46], it is also clear that intact mitochondrial structure is essential to regulate ROS imbalances [20]. Here, we suggest that while it appears that ROS generated from the electron transport chain generally decrease fission and fusion, the alteration of dynamics is dependent upon which mitochondrial dynamic is affected first by the ROS generator. For example, if fusion is increased, then the larger organelles might move slower, thus we see velocity is decreased as seen for HA or vice versa with rotenone and AMA. Or if velocity increases, it is logical to assume that the mitochondria are moving past each other too fast for fission and fusion to properly take place such as with AMA. Mammalian studies indicate that alterations of calcium levels will alter mitochondrial dynamics and an increase in fusion will increase ATP synthesis, a possible mechanism to overcome a decrease in ATP due to excessive ROS [27]. Additionally, it is known that fission and fusion are used to repair and maintain mitochondrial quality, thus changes to fission and fusion occur in response to ROS damage. Finally, it is important to note that *D. discoideum* cells have a high resistance to oxidative stress [25], and therefore all the aforementioned reasons may be occurring simultaneously, resulting in the various responses by mitochondrial dynamics in *D. discoideum.*

## 5. Conclusions

This study has shed light on the ROS-producing capabilities of a variety of chemicals on *D. discoideum*. Finding reliable ROS-stimulating compounds within model organisms is critical to furthering research on neurodegenerative diseases related to cellular oxidative stress, such as seeking to mitigate the effects of ROS accumulation on a cellular level.

Additionally, we show the effect of these various ROS generators on mitochondrial dynamics. Our work demonstrates that alteration of the microtubule cytoskeleton is not required to alter dynamics in response to ROS and it appears that ROS generated from the electron transport chain decrease fission and fusion. By understanding that different ROS alter mitochondrial dynamics in different ways, we know that more research is needed to better understand the effects of ROS on the cellular mechanisms that induce the pathogenesis of incurable neurodegenerative diseases. Further research in this field is needed with the hope that it will someday translate into developing new and more effective treatments for patients afflicted by neurodegenerative diseases.

## Data Availability

Data are contained within the article.
